# Editorial: Computational modeling for the assessment of the biomechanical properties of the healthy, diseased and treated spine

**DOI:** 10.3389/fbioe.2022.1016311

**Published:** 2022-10-11

**Authors:** Enrico Dall’Ara, Hendrik Schmidt, Marwan El-Rich, Grace D. O’Connell, Fabio Galbusera

**Affiliations:** ^1^ Department of Oncology and Metabolism, Mellanby Centre for Bone Research, University of Sheffield, Sheffield, United Kingdom; ^2^ INSIGNEO Institute for in Silico Medicine, University of Sheffield, Sheffield, United Kingdom; ^3^ Julius Wolff Institute, Charité—Universitätsmedizin Berlin, Corporate Member of Freie Universität Berlin and Humboldt-Universität zu Berlin, and Berlin Institute of Health, Berlin, Germany; ^4^ Healthcare Engineering Innovation Center, Department of Mechanical Engineering, Khalifa University, Abu Dhabi, United Arab Emirates; ^5^ Department of Mechanical Engineering, University of California-Berkeley, Berkeley, CA, United States; ^6^ Spine Center, Schulthess Clinic, Zürich, Switzerland

**Keywords:** spine, spinal fractures, posterior fixation, finite element, musculoskeletal model, implants

The human spine has been optimised through the evolutionary process resulting in a unique structural combination of hard (i.e., bone) and soft tissues (i.e., intervertebral disc, tendons, ligaments, cartilage). The spine and surrounding musculature provide flexibility and structural stability while subjected to loads of varying magnitudes and directions during daily activities and protect the spinal cord and nerve roots from excessive strain. These biological tissues have complex heterogeneous, anisotropic, nonlinear, and hierarchical properties, making their biomechanical characterization challenging. Moreover, aging, diseases, and injuries may affect biomechanical stability, leading to vertebral fractures and/or intervertebral disc (IVD) degeneration, which may induce pain and disability. Different interventions are available to fix or slow the damage progression of the affected tissues, from pharmacological and conservative treatments to surgeries. However, due to the complexity of the microstructure and material properties of the tissues that compose the spine, assessing disease progression or treatment effects on the spine’s biomechanical properties are not trivial.

Experimental assessments such as motion capture, complex mechanical loading using dedicated multi-axial rigs, strain analyses with strain gauges, and digital image or volume correlation have been used to characterize biomechanical properties of the spine at different dimensional levels, but they lack the flexibility of testing the same structure in different loading conditions until failure. Moreover, the intrinsic variability of the geometrical, structural, and material properties due to sex, age, size, disease progression, treatments, etc. makes it difficult to test all possible scenarios experimentally. In particular, it is very challenging to measure the influence of the properties of the microstructural components (e.g., remodelled bone, fibers in the annulus fibrosus) on bulk tissue material properties. Moreover, damage accumulation of these viscoelastic and poroelastic tissues with age or disease also contributes to experimental challenges in the characterisation of their material behaviour. Therefore, computational models of spine segments and entire lumbar, thoracolumbar, and cervical spines have been developed to evaluate the biomechanical properties of healthy and diseased spines (e.g., patients with osteoporosis, osteoarthritis, bone metastases, traumatic fractures), and to optimize spinal treatment. While generic or subject-specific computational models can be parameterized, efficiently test several loading conditions and comprehensively study the spine biomechanics, the experimental studies remain invaluable to inform, calibrate and validate the models. In fact, increasing the model credibility, based on model verification, validation, sensitivity analyses, and uncertainty quantification is fundamental for developing tools that can be used to support clinical decisions (The American Society of Mechanical Engineers (ASME), 2018).

The current Research Topic presents a unique collection of studies that increase our knowledge about spine biomechanics and stimulate discussions for the improvement of techniques used for computational model development and validation.

Four studies have used multi-body dynamics (MDB) models for evaluation of ROM, load distribution, muscle forces, and activation on both thoracolumbar and cervical spine. Müller et al. have used MBD models with subject-specific geometry to predict the load distribution in a healthy lumbar spine as function of the lordosis angle. They confirm earlier assumptions by Roussouly and Pinheiro-Franco (Roussouly and Pinheiro-Franco, 2011) that large lordosis angles generate more stress on the facet joints but less stress on the vertebral bodies and intervertebral discs. This result is important both for optimising therapeutic measures and for identifying boundary conditions for computational models at lower dimensional scales (e.g., Finite Element (FE) models). Alemi et al. have used inverse kinematics, informed from motion analyses of seven healthy participants, to evaluate the effect of different kinematic constraints on the performance of thoracolumbar spine MBD models. They concluded that kinematic constraints with 5 degrees of freedom was the best compromise to track measurements and produce smooth spine motion. Arshad et al. have developed an inverse dynamic model of the head-neck complex, including the head, C1-T1 vertebrae, and detailed soft tissues (517 nonlinear ligament fibers and 258 muscle fascicles). A comprehensive sensitivity analysis showed that increased segment mass led to increased disc loads and muscle activity, that disc stiffness affected only disc translation, and that by increasing muscle strength, the muscle activity largely decreased. These results show that these models can be used to study the effect of diseases and treatments, after appropriate model validation. Firouzabadi et al. have compared spine loads during static manual material handling activities for males and female subjects, using a whole body MBD model. Female subjects had larger compressive and shear loads when normalized to the body weight and larger forces in the oblique abdominal muscles, while male subjects had larger back extensor muscle forces. The study highlights the importance of considering sex-specific parameters.

Ten manuscripts have used detailed structural FE models to evaluate the mechanical properties of the IVD, single vertebra, or spine segments. While many FE models have been utilized to characterize biomechanical properties of vertebral bodies and spine segments, there are still challenges to accurately model of the IVD material, create efficient spinal segment models, and to using FE models predictions to optimise treatment strategies. Pickering et al. have developed a pipeline to create models of paediatric IVDs and performed a sensitivity analysis on the material model inputs. They found that IVD collagen fiber bundles are the main contributors of IVD mechanical behaviour and should therefore be integrated in patient specific FE models of the paediatric spine. In another study, Du et al. have created FE models of the IVD based on magnetic resonance imaging (MRI) data from bovine spine and evaluated the model sensitivity to different geometrical and material input parameters, highlighting that it is fundamental to model well the geometry of the vertebral endplates. It should be noted that while FE models have great potential in assessing the biomechanical behaviour of spine segments, process automation is needed to improve the efficiency and reduce operator dependency for clinical application. Caprara et al. have developed an automatic pipeline to create subject-specific FE models of the lumbar spine from CT images by using deep learning techniques to segment the geometries, statistical shape models to create the meshes, and FE models to simulate different loading conditions and predict ROM of the segments. This automatic tool, the results of which agreed with literature data, has the potential of improving the clinical applicability of biomechanical simulations. FE models have been widely used to test the effect of spine fixation for treating vertebral fractures, IVD degeneration, spine deformities and other diseases as well. Sensale et al. have performed a verification and sensitivity analysis for subject-specific FE models of a single vertebra implanted with two pedicle screws. They have reported that the diameter of the screw is more important than its length for minimising screw and bone deformations. Moreover, they highlighted the importance of modelling realistic screw geometry. Bereczki et al. have developed an L2-L4 spine segment FE model to study the stability of different implants for oblique lumbar interbody fusion when implanted in healthy or osteoporotic bone. They showed that spine segment stability was affected by the used implant and that osteoporosis increases the ROM for all tested constructs. Gierig et al. have used a large FE model of the lumbar spine that also included the pelvis and spinopelvic devices to study the best configuration in fixing spinopelvic fractures and to show its superiority compared to non-surgical treatment. Ke et al. have used an FE model of C2-T1 spine segments to evaluate different surgical treatments of adjacent diseased segment (ADS) after a primary anterior cervical discectomy and fusion (ACDF). The model suggests that a second ACDF leads to better outcomes compared to laminoplasty in the tested case. In another study, Wo et al. have used a combination of an FE model of C2-C7 cervical segment, wear tests and animal study on non-human primate, to study the biomechanics of cervical subtotal discectomy prosthesis (CSDP) as an alternative for ACDF. The FE models showed the influence of the implant position on the CSDP performance including ROM, bone-implant stress, and forces at the facet joints. Nikkhoo et al. have used poro-elastic FE models to study the biomechanical stability of rigid (Ti rods) or semi-rigid (PEEK) posterolateral fixation for ADS. They showed that, compared with Ti constructs, the PEEK prosthesis may be preferable as it is associated with a slightly higher ROM at the instrumented level and lower IVD height loss, fluid loss, axial stress, and collagen fiber strain in the adjacent disc. Tachi et al. have used an FE model of the spine of patients with adolescent idiopathic scoliosis to pre-plan the surgical correction procedure with pre-bent spinal rods. This preplanning system can be used to optimize which spine levels to instrument and the rod shape.

The above-mentioned studies showed how FE models can assist surgeons in identifying the best prosthesis in cases of different spine diseases. However, there are still challenges in creating more realistic computational models accounting for the hierarchical properties of the spine with multi-scale approaches, that for example, better estimate the loading scenarios or the effect of bone remodelling over time, and in validating the outputs of the models to improve their credibility and their future clinical applicability. Favier et al. coupled L1-L5 spine FE model with lower strain-driven algorithm to predict local bone changes induced by physiological loading conditions calculated from a previously developed full-body MBD model. They showed that in order to maintain trabecular and cortical bone health, a combination of moderate and more demanding activities (large spine movement and lifting tasks) are needed. In fact, moderate intensity activities alone were not found to be sufficient to maintain bone health in the vertebrae. Panico et al. have tested the effect of simulating realistic muscle forces in FE models of lumbar fixation implants. They coupled a previously developed MBD model of the thoraco-lumbar spine with articulated ribcage with a detailed FE model of T10-T12 segments to compare the results of models with realistic and simplified (pure moments) loading. Intact spine segments and instrumented spine segments with rods and screws were simulated. The realistic FE models showed similar ROM but higher stresses in the pedicle screws and in the posterior rods compared to the simplified models, showing the importance of using realistic loading when evaluating implant stresses. Pachocki et al. have used a global FE model that includes a concrete road safety barrier, an impacting vehicle, and an occupant. The occupant model includes a lumbar spine FE model to study the biomechanics of injuries during road barrier collision. The two-scales model estimated the loading condition on the spine model from a larger FE dynamic model of the subject in the impacting vehicle. They have shown that during the crash the loading on the lumbar spine is eccentric and leads to high axial loads and flexion bending moments on L1-L5, explaining why fractures are associated with this loading scenario.

Three manuscripts in the Research Topic have collected *ex vivo* or *in vivo* experimental data to inform and validate FE models of the IVD. Deneuville et al. have performed a proof-of-concept study using *ex vivo* MRI imaging of an L1-L3 ovine spine segment under different loading conditions before and after inducing damage of the IVD to evaluate its effect on the deformation of the nucleus pulposus. They combined this experiment with an FE model of the spine segments to evaluate the effect of the IVD damage on the stress field, showing the potential of this approach to study IVD biomechanics from MR images. Mengoni et al. have validated the outputs of MRI based FE models of the IVD, against *ex vivo* experiments performed on bovine specimens to measure the IVD bulge. The results showed that including subject-specific geometrical and material properties of the IVD in the FE models does not improve substantially the predictions of IVD stiffness and bulge. Finally, Zhou et al. Have developed a multiscale and multiphasic FE model of the IVD and validated it against experiments for the bovine caudal vertebra. In most cases the multiscale model, developed from experimental data of fiber and matrix mechanical behaviour, accurately predicted the structural response of the IVD, highlighting the importance of modelling the fibers, matrix-fiber interactions, and the fluid-based load bearing mechanism of this complex structure.

This Research Topic includes 20 peer-reviewed papers tackling different challenges in the topic of development and validation of computational modelling of spine biomechanics. Every paper reports the potential and current limitations of the developed approaches, highlighting progress that the research community has done in this area, and where we should focus to improve clinical applicability. We would like to emphasise that, while the presented research is fundamental for understanding the biomechanics of the spine and further develop computational models to support clinical decision making, a lot of work needs to be done to see these approaches used routinely. We anticipate that future studies that use biomechanical models of the human spine will be even more realistic and biofidelic (e.g., considering complex individualized loading and boundary conditions taking into account the everyday behaviour, functional behaviours and adaptations after surgical procedures or pain experiences, activation patterns, individualized material properties, *etc.*), more credible through comprehensive and systematic validation process using *ex vivo* and *in vivo* data, certifiable by regulatory bodies, adaptable to study a single subject or generic to study a patient population. To conclude, while there is no doubt that research in this exciting area will keep progressing and improving our knowledge of the Biomechanical Properties of the Healthy, Diseased and Treated Spine as demonstrated in this Research Topic, the research community should encourage and strengthen interdisciplinary collaborative research involving bioengineering, biology, and medicine.
